# Post-traumatic Arthritis of the Tarsometatarsal Joint Complex: A Case Report

**DOI:** 10.7759/cureus.923

**Published:** 2016-12-09

**Authors:** Chirag Kapoor, Amit Patel, Maulik Jhaveri, Paresh Golwala

**Affiliations:** 1 Orthopaedics, Sumandeep Vidyapeeth, Vadodara, Gujarat

**Keywords:** osteoarthritis, arthrodesis, tarsometatarsal joint

## Abstract

Tarsometatarsal (TMT) arthritis is characterized by instability and pain in the foot. The commonest cause is post-traumatic arthritis. A Lisfranc injury involves the articulation between the medial cuneiform and the base of the second metatarsal, which is considered a keystone to midfoot integrity. Neglected or undertreated injury to the Lisfranc joint complex leads to secondary arthritis and significant disability. We present a case of a young male patient with a two-year-old neglected Lisfranc joint injury and secondary osteoarthritis of the first, second, and fourth TMT joints, which we treated surgically with arthrodesis using screws, with a good functional outcome on final follow-up.

## Introduction

Tarsometatarsal (TMT) arthritis* *is characterized by midfoot instability, pain, and severe functional impairment. The most common cause is post-traumatic arthritis, followed by primary osteoarthritis and other inflammatory processes. Injury to the TMT joint complex occurs in one in 55,000 persons per year, which accounts for 0.2% of all fractures, and up to 20% of the cases are undiagnosed and left untreated [1].

The most common area of involvement is from Lisfranc injuries, resulting in degeneration of the first, second, and fourth TMT joints [2]. The Lisfranc joint injury pattern is notorious for developing secondary arthritis if left untreated or treated with incongruity. Joint destruction most typically develops over a long period secondary to biomechanical abnormalities [3]. In case of primary osteoarthritis, the second and third joints are most commonly involved [4].

We report a case of a young adult with a two-year-old Lisfranc injury that was treated conservatively and secondary osteoarthritis of the first, second, and fourth TMT joints, which was treated with arthrodesis using screws.

## Case presentation

A 32-year-old male patient presented to us with discomfort on walking and pain after prolonged standing on his right foot that had persisted for two years. The pain was aggravated by walking and climbing stairs. There was history of trauma to the same foot two years back when he had a Lisfranc injury, which was treated conservatively elsewhere. The patient had been taking analgesics intermittently since the last two years, but the pain was gradually increasing in intensity.

On examination of the foot there was tenderness over the first, second, and fourth TMT joints. There was no swelling and full range of foot and ankle movements. The arch of the foot was maintained. There was no deformity in the foot.

**Figure 1:**
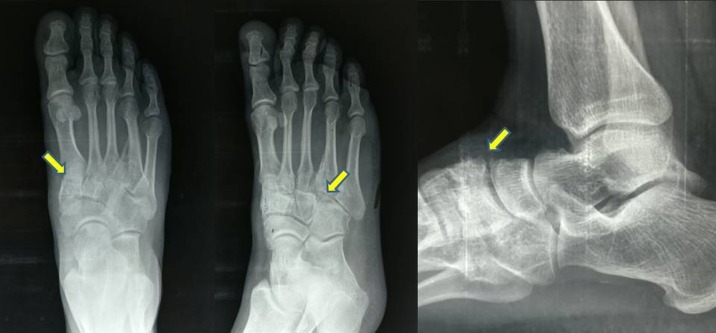
Pre-op radiograph (antero-posterior and lateral views) Shows arthritic first, second, and fourth TMT joints.

**Figure 2:**
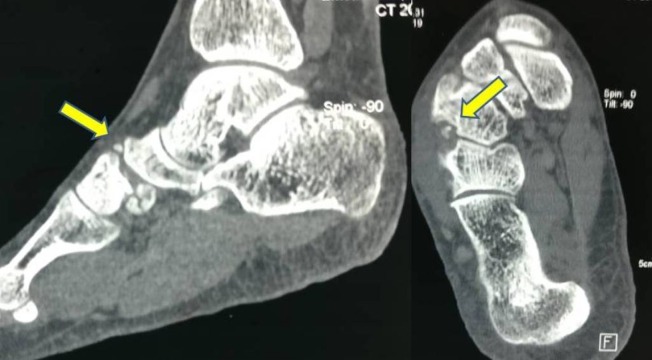
Computed tomography (CT) Shows arthritic first, second, and fourth TMT joints with osteophytes.

A weight-bearing antero-posterior (AP) radiograph showed incongruous first and second TMT joints with osteophytes formation with decrease in the joint space and marginal sclerosis of the joints. Lateral and oblique radiographs showed wide displacement of the second and fourth TMT joints with flattening of the longitudinal arch (Figure 1). A computed tomography (CT) showed incongruous joint surfaces with osteophytes around the first, second, and fourth TMT joints (Figure 2).

**Figure 3:**
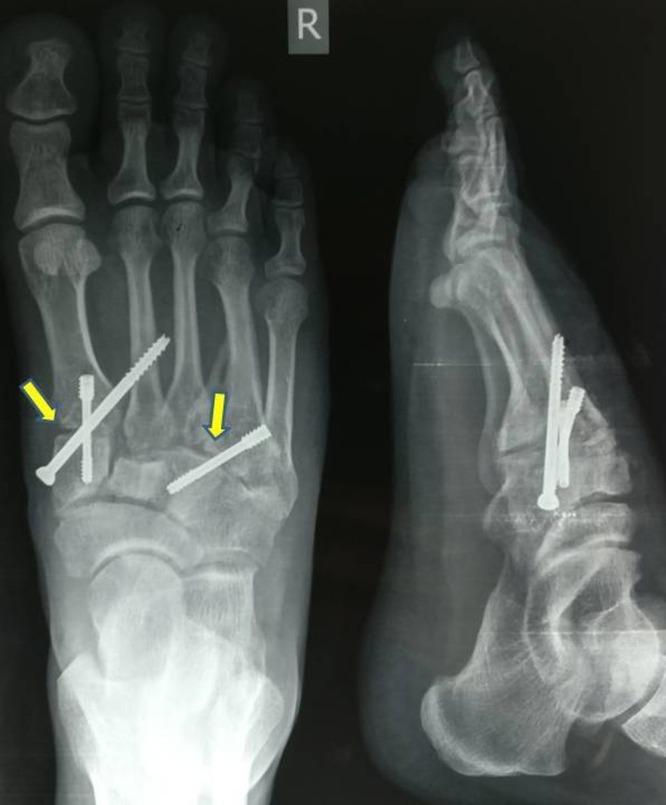
Post-op radiograph (antero-posterior and lateral views) Shows fixation of first, second, and fourth TMT joints with screws and presence of bone graft.

After obtaining a written informed consent, the patient was submitted for surgery. Two dorsal linear incisions over the foot were used; one between the first and second TMT joints and other over the fourth TMT joint. Arthritic joint surfaces of the first, second, and fourth TMT joints were shaved off till the subchondral bone and bone graft taken from the anterior iliac crest was put in the void created in the joints. The first and second TMT joints were fixed using two 3.5 mm cortical screws. One was passed from the first metatarsal into the medial cuneiform and the other was passed from the medial cuneiform to the second metatarsal (Figure 3). A third screw was passed from the base of the fourth metatarsal to the lateral cuneiform (Figure 3). The reduction and stability was assessed clinically and under an image intensifier during the procedure.

**Figure 4:**
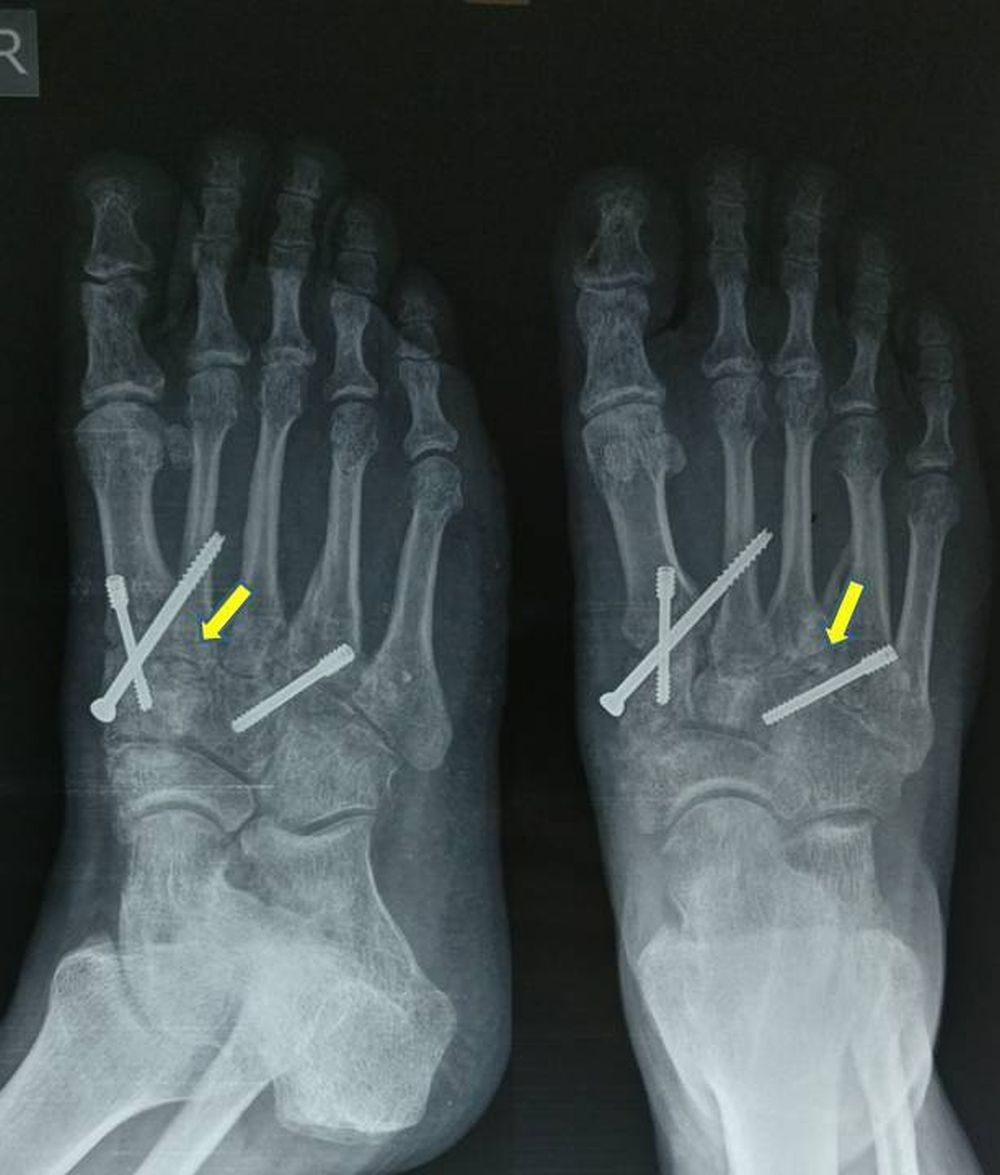
Follow-up radiographs at two months Shows fixation of first, second, and fourth TMT joints is well maintained after removal of the plaster cast.

**Figure 5:**
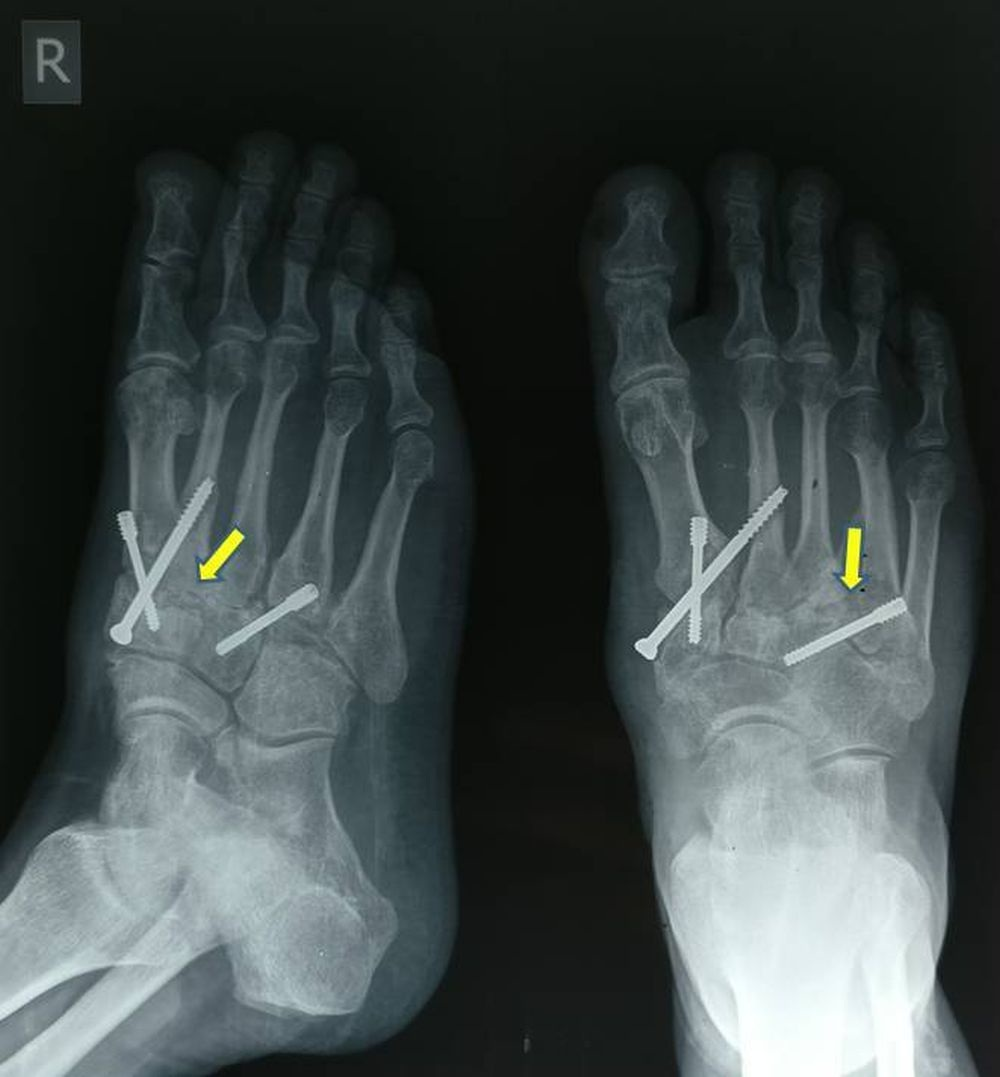
Final follow-up at 18 months Shows good fusion of the TMT joints with screws maintained in position.

Postoperatively, a below-knee plaster cast was given for a period of eight weeks and the patient was kept non-weight bearing. After two months, the cast was removed after radiological signs of fusion were seen (Figure 4). The patient was then allowed to bear full weight. At the 18-month follow-up, the patient was bearing full weight with no pain, and radiologically there was good fusion of the first, second, and fourth TMT joints (Figure 5).

## Discussion

Arthritis of the TMT joints is a challenging problem because of its high potential for chronic foot pain and functional disability. The primary aim of treatment is to afford pain relief by enhancing midfoot stability and modifying the loads sustained at the inflamed joints. Development of secondary degenerative changes is markedly increased if the extent of the injury has initially been unrecognised, if the injury has been only partially treated, or if the anatomy was not restored [5].

Nonsurgical options for treatment include non-steroidal anti-inflammatory drugs (NSAIDs), full-length rigid foot plates, and shoe modifications. If these methods fail to relieve symptoms to an acceptable level, arthrodesis of the painful tarsometatarsal joints is the treatment of choice [6]. The surgical goal is to reduce painful motion and increase the stability of the foot. This goal can be obtained through partial or total fusion of the tarsometatarsal joints. Correction of the deformity is a secondary concern. The decision to ascertain the extent of the arthrodesis should be based on the location of pain and the radiological appearance of the joints. Arthrodesis can be restricted to one joint or include multiple joints. The most common joint requiring stabilization is the first metatarso-cuneiform articulation [7].

Whether arthrodesis can be used in the fourth and fifth TMT joints continues to be controversial. Most authors approve the motion-preserving procedures since they believe that arthrodesis will increase the rate of non-union and stress fracture [8]. However, if gross pain, arthritic changes with obvious structural deformity or pseudoarthrosis is present in patients with malunion of the fourth and fifth TMT joints, arthrodesis may become an advisable option [8].

To expose the TMT joint complex, two dorsal incisions are usually taken. The first incision is centred over the second TMT joint and extensor hallucis longus (EHL) is retracted medially and the extensor digitorum brevis along with the neurovascular bundle is protected laterally. This gives access to the first and second TMT joints as well as the intercuneiform joints. The lateral incision is centred over the fourth TMT joint and the superficial branch of the peroneal nerve is at risk. This gives access to the third to fifth TMT joints [9].

Arthrodesis is indicated for minimal deformity with arthritis limited to the middle and/or medial column. Arthrodesis of the medial and middle joints improves stability and decreases pain with functional improvement [10].

## Conclusions

We conclude that Lisfranc injuries have to be treated appropriately as they can lead to debilitating secondary arthritis. If conservative treatment fails, arthrodesis is the option and multiple joints can be fused as required to gain optimal results, like in our case.
